# Mechanisms of Silique Dehiscence in Rapeseed: A Review of Research Progress

**DOI:** 10.3390/cimb47090755

**Published:** 2025-09-12

**Authors:** Menglin Zhou, Wuming Deng, Bingbing Dai, Qingqing Yu, Wei Zhou, Xiaofei Zan, Xi Song

**Affiliations:** 1 Nanchong Academy of Agricultural Sciences, Nanchong 637000, China; 2 College of Agronomy and Biotechnology, Southwest University, Chongqing 400715, China

**Keywords:** rapeseed, pod dehiscence, mechanization, genes, molecular mechanism

## Abstract

Silique dehiscence is a critical biological phenomenon in rapeseed production that significantly influences seed maturity, harvesting efficiency, and ultimately yield. As one of the world’s most important oilseed crops, studying the mechanisms underlying silique dehiscence in rapeseed (*Brassica napus* L.) not only aids in understanding fundamental principles of plant development but also provides a scientific basis for optimizing agricultural production practices. Silique dehiscence occurs naturally during the maturation process of rapeseed, with the timing and extent of this phenomenon directly affecting seed harvesting efficiency. This paper reviews the research progress regarding the mechanization of canola production, which enhances harvesting efficiency by enabling timely harvest coordination to minimize pre-harvest shattering losses and reduce post-harvest seed damage. Additionally, it addresses the factors influencing pod shattering, the process of pod shattering, the genes associated with this phenomenon, and the molecular mechanisms underlying pod shattering. These findings establish a foundation for a comprehensive understanding of pod shattering in canola.

## 1. Introduction

Rapeseed (*Brassica napus*) is one of the most important oilseed crops globally and occupies a crucial position in agricultural production. According to FAO data from 2025, global rapeseed production reached 85 million metric tons annually, with China contributing over 22% (19 million tons) of the total output, making it the world’s largest producer. Its economic value and multifunctionality establish it as a core crop in the agricultural production of many countries. Rapeseed not only provides over 15% of the global edible oil supply but also plays a significant role in the production of biofuels, accounting for 30% of the EU’s biodiesel feedstock, as well as serving as animal feed and industrial raw materials [1,2,3]. With the growth of the global population and the increasing prominence of food security issues, the high yield and sustainable cultivation of rapeseed have become particularly important [4,5,6]. Furthermore, rapeseed exhibits significant ecological benefits within crop rotation systems. Research has shown that rotating rapeseed with leguminous crops, such as soybeans, can markedly improve soil health and enhance the yield of subsequent crops. In the context of crop rotation systems, rapeseed serves as an excellent rotational partner for cereals, such as wheat, and legumes, including soybeans, due to its unique root exudates that suppress soil-borne pathogens. Research indicates that rotating rapeseed with soybeans can enhance subsequent crop yields by 12–18% through nitrogen fixation, as soybean roots contribute 50–100 kg N/ha. This rotation pattern not only improves land utilization efficiency but also reduces reliance on chemical fertilizers, leading to a decrease in nitrogen application rates by 20–30%, thereby aligning with the principles of sustainable agricultural development [7,8]. In addition, rapeseed exhibits strong adaptability to climate change, particularly demonstrating a certain level of tolerance to abiotic stresses such as high temperatures, drought, and salinity.

The splitting of siliques is a complex physiological process influenced by various factors, including cultivation factors, selection factors, source factors, genetic factors, physical mechanisms, and physiology. These factors interact to determine the mechanical strength and internal pressure of the silique, thereby affecting the frequency and severity of splitting. Firstly, cultivation factors play a significant role in silique splitting. Studies have shown that cultivation practices such as water management, fertilization strategies, and temperature regulation have a notable impact on the development and splitting of siliques. For instance, water stress significantly increases the risk of silique splitting, especially in the later stages of fruit development, when internal pressure exceeds the mechanical strength of the fruit skin, leading to splitting [9]. Furthermore, the application of calcium fertilizers has been proven to effectively enhance the mechanical strength of the fruit skin, reducing the occurrence of splitting [10]. These findings provide a theoretical basis for reducing silique splitting through optimized cultivation management. Selection factors and source factors also have a significant impact on silique splitting. Different varieties of rapeseed exhibit significant differences in sensitivity to silique splitting, which is closely related to their genetic background. For example, certain varieties demonstrate stronger resistance to splitting due to the structure of their fruit skin cell walls or the high expression of genes related to cell wall metabolism [11,12]. Additionally, source factors such as the genetic background of the seeds and their geographical adaptability can also influence the development and splitting of siliques. By selecting anti-splitting varieties and optimizing seed sources, the risk of silique splitting can be effectively reduced [13,14].

Genetic factors are one of the core determinants of pod shatter. Research indicates that pod shatter is closely related to the expression regulation of multiple genes, particularly those associated with cell wall metabolism, plant hormone signaling, and the transport of water and nutrients [15,16,17]. For instance, the expression levels of cell wall metabolism-related genes, such as polygalacturonase (PG) and expansin (EXP), are significantly higher in shatter-sensitive varieties compared to resistant varieties, which may lead to a reduction in the mechanical strength of the fruit skin [18]. Furthermore, the balance of plant hormones, such as auxin and abscisic acid, plays a crucial regulatory role in the development and shattering of pods [19]. By employing gene editing or molecular marker-assisted selection techniques, it is possible to screen and cultivate oilseed rape varieties resistant to shattering, providing a genetic solution to reduce pod shatter. The physical mechanism is a direct driving factor of pod shatter. Pod shatter typically occurs when the internal expansion pressure of the fruit skin exceeds its mechanical strength, a process influenced by the thickness of the fruit skin, the structure of the cell wall, and the distribution of internal water and nutrients [20]. For example, the expansion of fruit skin cells and the degradation of the cell wall can lead to a reduction in fruit skin strength, thereby increasing the risk of shattering [21]. Additionally, environmental factors such as changes in temperature and humidity can also affect the physical properties of the fruit skin, subsequently influencing the occurrence of shattering [22]. By studying the physical properties of pods and their interaction with environmental factors, scientific evidence can be provided for the development of cultivation and storage techniques that are resistant to shattering. Physiological factors also play a significant role in the cracking of pods. The development and cracking of pods are regulated by various physiological processes, including photosynthesis, respiration, and the accumulation of secondary metabolites [12]. For instance, carbohydrates produced during photosynthesis significantly influence the swelling of pods and the formation of internal pressure [23]. Additionally, the accumulation of secondary metabolites such as lignin and flavonoids can enhance the mechanical strength of the fruit skin, reducing the occurrence of cracking [24]. By regulating these physiological processes, the risk of pod cracking can be effectively minimized.

The degree of pod shattering is closely related to the maturity of rapeseed, particularly in the later stages of maturation, when the mechanical strength and crack resistance of the pods significantly decrease [25]. This shattering phenomenon is particularly severe under high temperatures, dry, and windy climatic conditions, potentially leading to yield losses of up to 8–12%, and in extreme cases, this proportion may rise to 50% [26]. The mechanical gradient of the siliques in different parts of the plant is significant: the siliques of the main stem exhibit a higher degree of lignification in the vascular bundles, with a fracture toughness that is 1.5 times greater than that of the lateral branch siliques [27,28]. This spatial mechanical difference arises from the gradient expression of the *BnSHP1* gene—its expression level in the main stem siliques is 3.2 times higher, maintaining cell wall integrity by inhibiting pectinase activity [29]. Phenotypic association analysis indicates that crack-resistant varieties form six to eight layers of thick-walled cells at a distance of 150 μm from the back seam, while susceptible varieties only form three to four layers, resulting in an increased local stress concentration coefficient. Spatial water dynamics show that the siliques at the apex have a thicker cuticular wax layer, leading to a 57% reduction in water evaporation rate compared to the base, thereby delaying the accumulation of elastic potential energy [30]. These cross-scale associations provide a biomechanical basis for designing spatially specific crack-resistant breeding strategies.

The impact of weather conditions on the cracking of rapeseed pods is a complex and multidimensional issue, involving the interaction of multiple factors such as genetics, environment, agronomic practices, and harvesting techniques. Research indicates that adverse weather conditions, such as strong winds, rainfall, and temperature fluctuations, significantly increase the risk of pod cracking, leading to yield losses [31]. Particularly during mechanical harvesting, the combined effects of weather factors and mechanical collisions can result in yield losses of up to 50%. This loss not only directly affects the economic benefits of rapeseed but also poses challenges to the sustainability of agricultural production. From a physiological mechanism perspective, weather conditions exacerbate the cracking phenomenon by influencing the physical structure and internal physiological processes of the pods. For instance, high humidity and rainfall can cause the pods to absorb water and swell, increasing their vulnerability, while strong winds may exert additional mechanical stress on the pods, further accelerating cracking. Additionally, temperature fluctuations may adversely affect the cell wall structure and lignification of the pods, reducing their crack resistance. These environmental factors interact with the genetic characteristics of rapeseed, making certain varieties more prone to cracking under specific weather conditions [25].

The yield loss caused by pod shattering and the challenges of seed cleaning during mechanical harvesting are critical issues that urgently need to be addressed in canola production. Research indicates that pod shattering not only directly affects the final yield of canola but also increases the difficulty of mechanical harvesting. This is particularly evident during the cleaning process, where scattered seeds mix with impurities, reducing cleaning efficiency and further impacting harvest quality [32]. Traditional cleaning equipment primarily relies on airflow and screens to separate seeds from impurities; however, due to the similar physical characteristics of scattered seeds and impurities, the cleaning efficiency remains low. To enhance cleaning efficiency, researchers have proposed a series of innovative designs. For instance, the adoption of a multi-stage cleaning system that combines airflow, vibrating screens, and photoelectric sorting technology can effectively improve the separation accuracy of seeds from impurities. Additionally, intelligent cleaning systems based on machine vision are gradually being applied in canola harvesting, achieving precise sorting by recognizing characteristics such as seed shape and color [33]. The implementation of these technologies not only enhances cleaning efficiency but also reduces seed loss, further improving the economic benefits of mechanical harvesting. By integrating variety improvement, cultivation management, and mechanical harvesting technologies, it is possible to effectively reduce yield losses caused by pod shattering and enhance the efficiency of mechanical harvesting.

Currently, the breeding of canola for crack resistance faces three major bottlenecks: The breakage of the phenotype–genotype association: Traditional rapeseed varieties are prone to pod shattering, resulting in a short harvest period and a mechanical harvest loss rate exceeding 30% [34]. Although there have been breakthroughs in developing shatter-resistant varieties, large-scale promotion still requires addressing the compatibility issues between varieties and agricultural machinery, such as pod strength and maturity consistency, to reduce mechanical harvest losses. Germplasm resources with excellent traits such as high yield, shatter resistance, and multiple disease resistance are relatively scarce, and the efficiency of genetic improvement needs to be enhanced. It is essential to explore shatter resistance-related genes in depth, accelerate the aggregation and transfer of quality genes, and cultivate varieties with superior comprehensive performance. The cultivation area for rapeseed is extensive, and ecological conditions vary significantly; thus, the adaptability of shatter-resistant varieties in different regions needs further validation. Additionally, supporting cultivation techniques (such as dense planting, fertilization, and pest and disease control) must be closely integrated with the characteristics of shatter-resistant varieties to fully realize their advantages [25]. This study aims to provide a theoretical framework and technical pathway for designing new rapeseed varieties with high crack resistance and suitability for mechanical harvesting by revealing the hydromechanical-genetic coupling mechanism.

## 2. The Importance of Mechanized Production to Improve the Yield of Rape

Mechanized production plays a crucial role in enhancing the yield of rapeseed, especially in the context of current global agricultural challenges such as labor shortages, rising production costs, and increasing environmental pressures. The mechanization of rapeseed production involves the comprehensive application of precision control equipment across five key stages: land preparation, precision sowing, field management, efficient harvesting, and post-processing, supported by a sensor-based monitoring system. This approach effectively addresses labor shortages and environmental pressures while optimizing resource efficiency [35]. Firstly, mechanized production enables precise sowing and fertilization, ensuring that rapeseed plants develop under optimal growth conditions. For instance, the application of double-season direct seeding technology, such as the rotation of cotton and rapeseed, not only reduces manual labor but also improves land utilization by optimizing nitrogen fertilizer usage, thereby significantly increasing yield and economic benefits. Furthermore, mechanized sowing and fertilization can reduce the waste of chemical fertilizers and pesticides, lower environmental pollution, and align with the development goals of sustainable agriculture [36,37].

The improvement of mechanized harvesting technology is crucial for enhancing rapeseed yield. During the maturation period, rapeseed is prone to pod shattering, which can result in yield losses of up to 8–12%, and under adverse weather conditions, this loss may reach as high as 50% []. By developing combine harvesters suitable for rapeseed harvesting and optimizing their design and structure, it is possible to effectively reduce losses caused by pod shattering and mechanical collisions. Furthermore, mechanized harvesting can shorten the harvesting time, thus avoiding yield losses due to delayed harvesting, especially in large-scale planting areas where the advantages of mechanized harvesting are particularly significant [38]. Further research indicates that selecting dwarf and compact rapeseed varieties suitable for mechanized harvesting can significantly improve harvesting efficiency and reduce losses. For instance, the discovery and application of the dwarf gene BnaC04.BIL1 not only reduced plant height but also enhanced lodging resistance, making it more suitable for mechanized harvesting [39].

Moreover, mechanized production has facilitated an increase in the planting density of rapeseed. The cultivation of dwarf and semi-dwarf rapeseed varieties, such as the sdA03 mutant, has not only increased planting density but also enhanced photosynthetic efficiency and nutrient utilization by optimizing plant structure, thereby significantly improving yield per unit area [40]. The promotion and application of these varieties, combined with mechanized sowing and harvesting technologies, can further unleash their yield potential. At the same time, mechanized production has also driven the intensification and scaling of rapeseed cultivation, particularly in major rapeseed-producing areas like China, where the application of mechanization technology has significantly improved production efficiency, reduced production costs, and consequently increased farmers’ income and planting enthusiasm [41].

It is noteworthy that mechanized production has also promoted the sustainable development of rapeseed cultivation. By optimizing energy utilization efficiency in mechanized production, such as reducing diesel consumption and improving operational efficiency, significant reductions in greenhouse gas emissions in agricultural production can be achieved [42]. Furthermore, mechanized production can facilitate the resource utilization of agricultural waste, such as the application of straw return technology, which not only enhances soil fertility but also reduces environmental pollution [43]. The implementation of these measures not only contributes to the stable growth of rapeseed yield but also provides important support for the sustainable development of global agriculture [41]. The further expansion of rape planting benefits can be achieved through significant technological innovations. These innovations include harvest synchronization, where the coupling of the heat accumulation model with pod moisture sensors has increased the timely harvest rate by 40%. Additionally, the CRISPR-edited *BnSHP1* mutant has effectively reduced field loss from 12% to 5% by delaying the crack angle [29]. Another key innovation is system integration, which combines Internet of Things machinery for real-time yield monitoring with semi-dwarf cultivars (30–150 cm plant height adaptability). Future advancements will focus on AI-powered decision support systems that synchronize harvest timing with pod dehiscence thresholds [44,45].

## 3. Effect of Pod Dehiscence on Rapeseed Production

The impact of pod cracking on rapeseed production is a research topic of significant interest in the fields of agricultural science and plant physiology. Pod dehiscence poses a significant threat to global rapeseed production, with direct yield losses averaging between 8% and 12% in major producing regions, such as China and the European Union. Mechanistically, this phenomenon involves lignification-induced degradation of the cell wall at the dorsal suture, which results in a reduction in pod wall strength by 40% to 60% during late maturation. Field studies have confirmed that wind speeds exceeding 6 m/s can increase shattering rates by a factor of 3.5, while improper mechanical harvesting can exacerbate losses to over 20% in susceptible varieties [34,46,47]. As an important reproductive organ of rapeseed, pod cracking not only directly affects the harvesting efficiency of seeds but may also have profound implications for the overall yield and quality of the crop.

From a molecular biology perspective, the regulation of pod shattering involves the interaction of various genes and hormones. Moreover, pod shattering is closely related to the genetic characteristics of different rapeseed varieties. Studies have shown that certain rapeseed varieties exhibit lower shattering rates due to the higher mechanical strength of their pod walls, providing a theoretical basis for breeding crack-resistant varieties through selective breeding [34,48]. Therefore, in-depth research on the molecular mechanisms and genetic basis of pod shattering is of great significance for the development of crack-resistant rapeseed varieties.

From the perspective of agricultural practices, the impact of pod shattering on canola production is not only reflected in yield loss but also involves harvesting costs and seed quality. Due to seed loss caused by shattering, farmers often need to increase harvesting frequency or adopt more refined harvesting techniques, which not only raises production costs but may also adversely affect soil structure and subsequent crop planting [49]. Moreover, scattered seeds in the field are susceptible to pest and disease infestations, which reduces seed quality and storage stability, thereby affecting the economic value of canola [50]. Therefore, developing effective field management strategies, such as optimizing harvest timing, improving harvesting machinery, and using chemical regulators to delay pod shattering, has become an important research direction for reducing yield losses and enhancing the efficiency of canola production [51,52].

In the context of sustainable development, the issue of pod shattering is closely related to resource utilization efficiency and environmentally friendly agriculture. For instance, improving the pod-shattering resistance of rapeseed varieties through biotechnological means can reduce reliance on chemical regulators, thereby diminishing the environmental impact of agricultural production [53]. Furthermore, the seed dispersal caused by pod shattering may also affect field biodiversity, as the dispersed seeds could become a breeding ground for weeds or pests, thus increasing the difficulty of field management [54]. Therefore, when formulating rapeseed production strategies, it is essential to consider not only yield and economic benefits but also ecological balance and sustainable resource utilization.

The impact of pod shatter on rapeseed production is a multifaceted issue that encompasses various fields such as plant physiology, genetics, agricultural engineering, and environmental science. By conducting in-depth research on the mechanisms and regulatory pathways of pod shatter, and integrating breeding technologies with field management practices, it is possible to effectively reduce yield losses and enhance the sustainability and economic viability of rapeseed production. Future research should further explore the molecular regulatory networks of pod shatter and develop efficient and environmentally friendly anti-shatter technologies to provide scientific support for the healthy development of the rapeseed industry.

## 4. The Cracking Process of Rape Pods

Rapeseed (*Brassica napus* L.) is one of the world’s important oilseed crops, and the process of pod shattering is a key factor affecting seed yield and quality. Pod shattering mainly occurs during the maturation stage, where the loss of moisture from the pod wall leads to the separation of the cell layers between the pod wall and the septum, subsequently triggering pod shattering []. This process involves the collapse of the cell wall, the degradation of the middle lamella, and the enhanced activity of hydrolytic enzymes [55]. Pod shattering not only results in seed loss but also increases the difficulty of mechanical harvesting, particularly in developing countries, where this issue is especially pronounced due to the lack of efficient harvesting equipment []. Therefore, studying the molecular mechanisms and regulatory pathways of pod shattering in rapeseed is of significant importance for enhancing rapeseed yield and economic benefits.

The siliques of *Brassica napus* consist of two valves and a replum. During the dehiscence process, the dehiscence zone and the inner epidermis layer b play crucial roles. The dehiscence zone is located at the junction of the replum and the valves, comprising a lignified layer and a separation layer. The lignified layer is situated on the valve side, with cells that are highly lignified; the separation layer is on the replum side and consists of non-lignified cells. The inner epidermis layer b is located on the inner side of the valves and is composed of a layer of highly lignified cells [56,57]. Upon maturation of the silique, both the lignified layer of the dehiscence zone and the inner epidermis layer b have completed their lignification process, collectively forming the rigid framework of the valves. Due to the dehydration shrinkage of the mesocarp and exocarp of the valves, the framework formed by the lignified layer of the dehiscence zone and the inner epidermis layer b deforms towards the exocarp side, accumulating force that detaches from the replum. Meanwhile, the separation layer of the dehiscence zone is affected by relevant pectinase, weakening the intercellular connections. Ultimately, when the force accumulated by the valves that detaches from the replum exceeds the adhesion force between the cells of the separation layer, it prompts the separation of the valve from the replum at the dehiscence zone’s separation layer, leading to the opening of the entire silique [27,].

**Figure 1:**
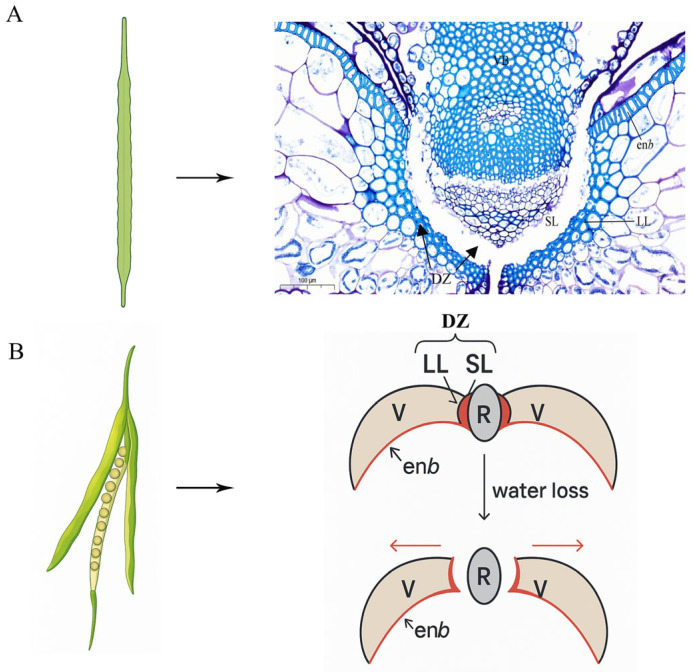
Morphological composition and cracking process of pods in *Brassica napus*. (**A**) Paraffin sections of mature pods (cross-section). (**B**) Cracking model of pods. Red arrows indicate the direction of force. DZ, departure zone; enb, endodermis b; LL, lignified layer; R, embryo frame; SL, separation layer; V, fruit petals; VB, vascular bundle. Scale bars: 100 μm.

Pod shattering in *Brassica napus* initiates at physiological maturity, characterized by seed moisture levels around 45%, through a three-phase biomechanical process: Phase I: Dehydration-triggered stress, The loss of pod wall moisture from 85% to 35% induces tensile stress exceeding 2.5 MPa along the dorsal suture, resulting in microfractures in 60–70% of vascular bundles within 72 h. Phase II: Cell wall disassembly—localized hydrolysis of middle lamella pectins by polygalacturonase, which peaks at an activity of 120 U/g FW, weakens the dehiscence zone (DZ). Concurrently, lignin deposition in endocarp b cells, reaching up to 28% of dry weight, creates differential shrinkage forces. Phase III: Mechanical separation—as moisture levels drop below 20%, cellulose microfibril reorientation from 35° to 55° facilitates layer separation (Figure 1) [27,58].

The process of pod dehiscence in rapeseed is a complex biological phenomenon that involves various factors, including genetics, physiology, and biochemistry. A deeper investigation into the molecular mechanisms underlying this process will not only help to reveal the universal laws governing pod dehiscence in plants but also provide new ideas and methods for the genetic improvement of rapeseed and other oilseed crops [59].

## 5. Research Progress on Silique Dehiscence Gene

Pod dehiscence is a critical step in the reproductive process of plants, and its regulatory mechanisms involve the coordinated action of multiple genes. In recent years, with the rapid development of molecular biology techniques, researchers have gained a deeper understanding of the functions of pod dehiscence genes and their regulatory networks.

The essence of the cracking of rapeseed siliques originates from a synergistic failure mechanism that encompasses hydraulics, mechanics, and genetics. The primary inducing factors include hydraulic stress gradients, where cells in the cracking zone (DZ) preferentially lose water through aquaporins, creating osmotic pressure differences that trigger localized stress. Additionally, localized cell wall remodeling occurs as pectinase degrades high-ester pectin into low-ester forms, which reduces intercellular adhesion, while lignin nanopores exacerbate stress concentration [60]. The prevention and control mechanisms are realized through gene-environment interactions: CRISPR editing of *BnSHP1* delays programmed cell death, reducing field loss rates from 12% to 4.7%; intelligent water regulation combined with cuticle thickening can increase the hydraulic stress threshold by 1.8 times [29]. Multi-scale analysis provides precise targets for synchronously blocking the causal chain of cracking.

At the molecular mechanism level, the cracking of silique involves the regulation of multiple key genes. For instance, the *SHATTERPROOF* (*SHP*) gene family has been found to play a decisive role in silique cracking in *Arabidopsis* [61]. The *SHP* gene encodes a MADS-box transcription factor that promotes the differentiation and degradation of silique wall cells by regulating the expression of downstream target genes [29]. Additionally, the *INDEHISCENT* (*IND*) gene has also been shown to play an important role in the process of silique cracking; the bHLH transcription factor it encodes works in conjunction with the SHP gene to regulate the activity of cell wall degrading enzymes [62]. Notably, the expression of these genes is finely regulated by plant hormone signals, with gibberellin inhibiting the expression of *SHP* and *IND* through DELLA proteins, while abscisic acid enhances their expression via transcription factors such as *ABI5* [63,64]. Genes and hormones influence the physical and chemical parameters of cell walls by altering the spatial distribution of cell wall components, the kinetics of water transport, and the temporal patterns of enzyme activity. This leads to a significant reduction in cell wall stiffness in the DZ region, a marked decrease in intercellular adhesion, and a local stress concentration coefficient that exceeds the critical threshold, ultimately resulting in the cracking of the locule.

In addition to the regulation by hormones and transcription factors, cell wall degrading enzymes also play a crucial role in the dehiscence of siliques. The double mutant *adpg1adpg2* of the pectinase synthesis genes *ADPG1* and *ADPG2* enhances the resistance of *Arabidopsis thaliana* to fruit cracking. *ADPG1/2* is located downstream of *IND*, suggesting that pectinase may be the most downstream factor in the regulation network of fruit cracking [65,66]. Transgenic plants with silenced expression of the hemicellulase gene *MANNANASE7* exhibit enhanced resistance to pod splitting []. In mustard, the overexpression of *FUL* can lead to the impaired development of the silique region, resulting in the formation of non-dehiscent siliques [67]. Knockout of the gibberellin synthesis gene *BnGA4s* in *Brassica napus* not only results in reduced plant height but also affects the development of the abscission layer in the pod, enhancing the resistance of the rapeseed pods to splitting [68]. Utilizing gene editing methods to knock out *BnALC* in *Brassica napus* can significantly enhance the resistance of rapeseed to pod shattering [69].

In recent years, with the rapid development of biotechnology, researchers have gained a more comprehensive understanding of the regulatory network of pod shattering genes. For instance, through RNA sequencing technology, researchers have identified several novel genes associated with pod dehiscence, including those encoding cell wall modifying proteins and signaling factors [70,71]. Furthermore, it has been demonstrated that epigenetic regulation plays a crucial role in the process of pod dehiscence, where DNA methylation and histone modifications regulate the spatiotemporal specificity of pod dehiscence by influencing the expression of key genes [72]. The regulatory mechanisms of transcription are also crucial for the fine control of gene expression.

Despite significant progress in the study of pod shattering genes, many questions remain to be addressed. For instance, do functional differences exist in pod shattering genes among different species? How do environmental factors influence the expression of pod shattering genes? These questions necessitate further exploration through interdisciplinary research. Future studies could integrate genome editing, single-cell sequencing, and systems biology techniques to elucidate the molecular mechanisms underlying pod shattering, thereby providing theoretical support for crop improvement and agricultural production [73,74].

## 6. Molecular Mechanism of Rapeseed Pod Dehiscence

### 6.1. Regulation Network of Pod Abscission Zone Development in Rapeseed

The development regulatory network of the silique abscission zone is a key biological process affecting seed yield and mechanical harvesting efficiency in rapeseed (*Brassica napus*). In recent years, with advancements in molecular biology and genomics technologies, researchers have gradually revealed the key genes and regulatory networks involved in this process, providing new theoretical foundations and technical support for rapeseed breeding. First, the core mechanism of silique abscission zone development involves the regulation of cell wall degrading enzymes.

Furthermore, hormonal signaling pathways play a crucial role in the development of the abscission zone in fruits. Jasmonic acid (JA) and auxin are key hormones that regulate the development of the abscission zone. Research has shown that the *TIFY/JAZ* gene family plays a central role in JA signaling, particularly under high-temperature stress, where the expression of these genes is significantly upregulated during the development of the abscission zone [75]. Additionally, key transcription factors in the auxin signaling pathway, such as *BZIP11*, have been shown to exert regulatory effects during early seed development, with their expression levels directly influencing the formation of the abscission zone [76]. These studies reveal the complex interactions between hormonal signals and the development of the abscission zone, providing important clues for further elucidating the regulatory network.

Transcription factor networks also play a crucial role in the developmental regulation of the silique abscission zone. For instance, *WRINKLED1* (*WRI1*) and *LAFL* (which includes *LEC1*, *ABI3*, *FUS3*, and *LEC2*) transcription factors are key players in seed development and oil biosynthesis, and they may also indirectly influence the development of the silique abscission zone. Through genome-wide association studies (GWAS) and RNA sequencing technologies, researchers have identified a series of candidate genes associated with the expression of *WRI1* and *LAFL*, which may participate in the development of the silique abscission zone by regulating the expression of cell wall degrading enzymes or hormone signaling pathways. Additionally, the *MYB56* transcription factor has been shown to indirectly affect seed oil accumulation and the development of the silique abscission zone by regulating the expression of *BnaA09.LEC1* [77]. The role of epigenetic regulation in the development of the silique abscission zone is also gradually being revealed. DNA methylation and small interfering RNA (siRNA) exhibit significant subgenomic bias during the development of the silique abscission zone, particularly with higher methylation levels in the Cn subgenome, which may influence the formation of the silique abscission zone by regulating gene expression. Furthermore, the expression patterns of siRNAs show conservation during the development of the silique abscission zone, indicating their important role in the regulatory network [78].

The regulatory network of pod development in rapeseed is a multi-layered and complex system that involves various aspects such as cell wall degradation, hormone signaling, transcription factor networks, and epigenetic regulation. By analyzing this network, researchers can not only gain a deeper understanding of the molecular mechanisms underlying pod development but also provide new targets and strategies for rapeseed breeding. For example, regulating the expression of key genes through gene editing technologies (such as CRISPR/Cas9) can significantly enhance the crack resistance of pods, thereby improving the efficiency of mechanical harvesting [79]. Moreover, by integrating transcriptomics and metabolomics analyses, key metabolic pathways and regulatory nodes during pod development can be further revealed, providing theoretical support for the improvement of rapeseed yield and quality [80,81]. In the future, with the further development of multi-omics technologies, research on the regulatory network of pod development in rapeseed will become more in-depth, leading to greater breakthroughs in rapeseed breeding and agricultural production.

### 6.2. Development of Lignified Tissue of Rape Silique

The development of lignified tissues in the siliques of rapeseed (*Brassica napus* L.) is a crucial biological process in the plant’s life cycle, directly affecting the mechanical strength of the siliques, seed release, and stress resistance. Lignification, as a complex cell wall modification process, is primarily regulated by the phenylpropanoid metabolic pathway, involving the synergistic action of various enzymes and transcription factors. Studies have shown that lignification significantly intensifies during specific stages of silique development, particularly in the late maturation phase, where the degree of lignification directly influences the silique’s cracking and seed dispersal efficiency. In the lignification process of the silique, the lignification of the endocarp b layer cells is particularly critical, as the thickening of their cell walls and the deposition of lignin provide necessary mechanical support to the silique, preventing premature cracking [11,82]. Furthermore, research has also found that the degree of lignification is closely related to the silique’s disease resistance and stress tolerance, especially in resisting infections from pathogens such as *Sclerotinia sclerotiorum*, where the formation of lignified tissues can effectively restrict the spread of pathogens [83].

At the molecular mechanism level, the regulation of lignification in *Brassica napus* pods involves various transcription factors and metabolic pathways. For instance, the MYB transcription factor family plays a crucial role in the lignification process, with *BnMYB43* and *BnMYB69* directly influencing the deposition of lignin and the composition of the cell wall by regulating the expression of lignin biosynthesis-related genes [84,85]. Furthermore, Cinnamoyl-CoA reductase (CCR), a key enzyme in the lignin biosynthetic pathway, shows a positive correlation between its expression level and the degree of lignification. Overexpression of the *CCR* gene can significantly enhance the lignin content in pods, thereby improving their mechanical strength and disease resistance [83,86]. These findings provide an important theoretical basis for the genetic engineering of pod lignification.

The development of lignified tissues in *Brassica napus* is a complex biological process that involves the regulation of various molecular mechanisms and environmental factors. A thorough investigation into the molecular basis of lignification and its regulatory network not only aids in revealing the biological principles governing plant cell wall modifications but also provides significant theoretical foundations and technical support for rapeseed breeding and agricultural production. Future research can further explore the interactions between lignification and other biological processes, such as cell division and hormone signal transduction, as well as the precise regulation of lignification levels through gene editing technologies, paving new pathways for the genetic improvement and sustainable development of rapeseed.

### 6.3. Rape Pod Cell Wall Modification Related Enzymes

In recent years, the role of cell wall-modifying enzymes in regulating the process of dehiscence in siliques has gradually become a research hotspot. The changes in the composition and structure of the cell wall have a decisive impact on silique dehiscence, and the activity regulation of the associated enzymes is one of the core mechanisms for achieving this process. In the later stages of silique development, the degradation and reorganization of the cell wall are crucial steps leading to silique dehiscence. This process is primarily accomplished through the synergistic action of various cell wall-modifying enzymes, including pectin methylesterase (PME), cellulase (CE), polygalacturonase (PG), and β-galactosidase (β-Gal) [87]. PME influences the rigidity and plasticity of the cell wall by regulating the methylation degree of pectin, thereby providing the necessary mechanical support for silique opening [88]. Furthermore, cellulase and polygalacturonase promote the relaxation and rupture of the cell wall by degrading cellulose and pectic polysaccharides within the cell wall [89]. β-galactosidase plays a role in the hydrolysis of galactosidic bonds, participating in the degradation and remodeling of cell wall polysaccharides [87].

Research indicates that the expression and activity of cell wall-modifying enzymes during the dehiscence of *Brassica napus* pods are regulated by various internal and external factors. For instance, hormone signaling pathways, such as jasmonic acid and ethylene, play a crucial role in pod dehiscence by modulating the gene expression of relevant enzymes, thereby affecting the degradation and remodeling of the cell wall [90]. Additionally, environmental factors such as temperature, humidity, and light may also significantly impact the pod dehiscence process by influencing enzyme activity or gene expression. For example, high-temperature stress may lead to a reduction in the activity of cell wall-modifying enzymes, consequently inhibiting pod dehiscence [91].

In terms of molecular mechanisms, the study of gene expression and functions of enzymes related to pod dehiscence has provided important clues for revealing the regulatory network of this process. For instance, transcriptome analysis indicates that at the critical stage of pod dehiscence, the gene expression of various cell wall-modifying enzymes is significantly upregulated, including PME, CE, PG, and β-Gal [90]. Furthermore, protein interaction network analysis reveals the complex regulatory relationships between these enzymes, laying the foundation for further elucidation of the molecular mechanisms of pod dehiscence. For example, PME inhibitors (PMEIs) regulate the methylation status of pectin by interacting with PME, thereby affecting the mechanical properties of the cell wall [88].

In applied aspects, regulating the activity of cell wall modification enzymes provides a potential strategy for improving the efficiency of pod dehiscence and seed yield in *Brassica napus*. For instance, targeted regulation of the gene expression of key enzymes such as PME or PG through gene editing technologies (e.g., CRISPR/Cas9) may enable precise control over the pod dehiscence process [88]. Additionally, the application of exogenous hormones or chemicals could optimize the timing and extent of pod dehiscence by modulating enzyme activity [91]. For example, the application of jasmonic acid and ethylene has been shown to enhance the expression and activity of enzymes related to pod dehiscence, thereby increasing seed yield [90].

### 6.4. Effect of Hormones on Pod Dehiscence in Rapeseed

Hormones play a crucial role in the growth and development of plants, particularly in regulating physiological processes such as fruit cracking. Recent studies have shown that plant hormones play an important role in regulating pod cracking, especially the interactions between hormones such as Indole-3-acetic acid (IAA), Gibberellins (GA), Abscisic acid (ABA), and Ethylene (ET), which have a significant impact on pod development and cracking [92,93].

Firstly, indole-3-acetic acid (IAA) influences the morphology of siliques during the early stages of development by regulating cell division and expansion. Studies have shown that genes associated with the auxin signaling pathway (such as *BnaA7.ARF17*) are significantly expressed during silique development, with expression levels closely related to silique length and degree of dehiscence [92]. Furthermore, auxin also affects the mechanical strength of the silique walls by regulating the biosynthesis of cellulose and lignin, thereby indirectly influencing dehiscence behavior. Gibberellins (GA), on the other hand, play a crucial role during the later stages of silique development, promoting cell expansion and degradation of the silique walls. Research has found that overexpression of the GA 2-oxidase gene (*BnGA2ox6*) inhibits silique growth but does not significantly affect seed development, indicating that GA’s regulation of silique dehiscence may be achieved through the modulation of cell wall degrading enzyme activity in the silique walls [93].

Abscisic acid (ABA) and ethylene (ET) are more involved in the final triggering stage of pod splitting. ABA promotes the senescence and cracking of the pod wall by regulating the expression of genes related to cell wall degradation [92]. Ethylene is considered a direct trigger of pod splitting, as it activates the activity of cell wall degrading enzymes, such as pectinase and cellulase, leading to a reduction in the mechanical strength of the pod wall and ultimately resulting in cracking [94]. Furthermore, genes associated with the ethylene signaling pathway, such as ERF, are significantly upregulated during the late stages of pod development, further supporting their critical role in the cracking process [95].

In addition to the direct effects of the aforementioned hormones, the interactions between hormones also play a significant role in the cracking of silique. For instance, the synergistic effect of auxin and gibberellin can promote the cellular expansion of the silique wall, while the antagonistic effects of ABA and ethylene regulate the timing of silique cracking [92]. Furthermore, the cross-regulation of hormone signaling pathways with other metabolic pathways, such as phenylpropanoid metabolism and cell wall biosynthesis, further complicates the regulation of silique cracking [94]. Transcriptome analysis and gene co-expression network analysis (WGCNA) revealed the roles of several key genes and metabolic pathways associated with hormone signaling pathways in pod dehiscence. For instance, genes related to starch and sucrose metabolism, photosynthesis, and secondary cell wall biosynthesis were significantly expressed during pod development, and these metabolic pathways interact with hormone signaling pathways to jointly regulate pod dehiscence [96]. Additionally, miRNA-mediated gene regulation also plays a crucial role in hormone signal transduction; for example, bna-miR164 and bna-miR172 target and regulate *BnaA2.PDF2.5* and *BnaC7.PDF2.6*, respectively, and the expression changes in these genes may affect the final output of hormone signaling pathways [97].

**Figure 2:**
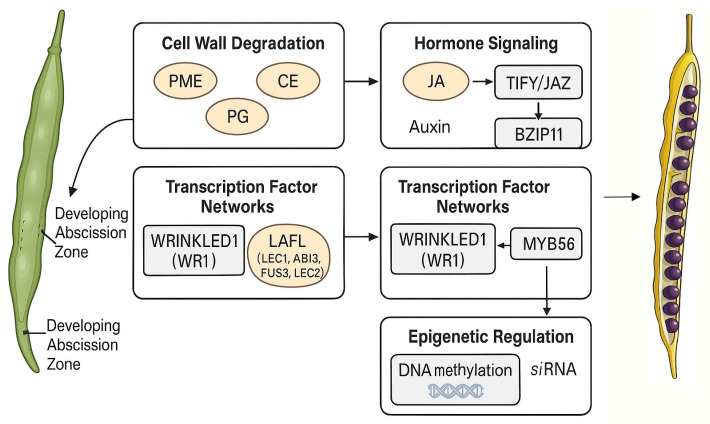
Factors affecting pod dehiscence in rapeseed. PME, pectin methylesterase; CE, cellulase; PG, polygalacturonase; JA, jasmonic acid; *LAFL*, transcription factors including *LEC1*, *ABI3*, *FUS3* and *LEC2*.

Hormones play a complex role in the regulation of silique dehiscence in *Brassica napus*, involving multiple levels and pathways. Future research could further explore the functions of hormone signaling pathways and their associated genes related to silique dehiscence. By employing gene editing and molecular breeding techniques, it may be possible to optimize the hormonal regulatory network, leading to the cultivation of new varieties of rapeseed that are high yielding and resistant to dehiscence. This research not only holds significant theoretical implications but also provides new directions for the practical application of rapeseed production (Figure 2) [92,94].

The core mechanism of pod shattering in rapeseed involves cell wall degradation, lignification regulation, and a complex feedback mechanism of gene networks. By regulating the expression of key genes through gene editing technology, combined with optimizing mechanical harvesting processes, it is possible to effectively enhance the resistance of rapeseed pods to shattering, thereby reducing yield loss and improving the efficiency of mechanical harvesting [98]. These studies provide important genetic resources and technical support for rapeseed breeding practices, holding significant agricultural application value.

## 7. Conclusions and Perspectives

In recent years, significant progress has been made in the study of the mechanisms underlying the pod shatter of rapeseed, revealing the critical roles of multiple genes and molecular pathways in regulating pod shattering. Through gene editing technologies such as CRISPR/Cas9 and traditional breeding methods, researchers have successfully identified several genes associated with pod shattering and developed rapeseed varieties with enhanced shatter resistance. The mechanisms of pod shattering in rapeseed involve a complex interplay of molecular regulatory networks, morphological traits, and mechanical properties. The use of gene editing technologies, genetic analysis, and mechanical optimization provides a scientific basis for developing shatter-resistant rapeseed varieties and improving rapeseed yield.

The splitting of rapeseed pods is one of the key issues affecting rapeseed yield and harvesting efficiency. Particularly during mechanical harvesting, the seed loss caused by pod splitting severely restricts the economic benefits of rapeseed production. In recent years, with the deepening of research in genome editing technology, molecular biology, and biomechanics, significant progress has been made in understanding the mechanisms behind rapeseed pod splitting. However, many critical issues still need to be addressed in the future.

Firstly, in terms of molecular mechanisms, although previous studies have indicated that several genes (such as *BnSHP1*, *BnSHP2*, *BnJAG*, etc.) play important roles in regulating silique dehiscence, the specific functions of these genes and their expression patterns during different developmental stages of canola still require further elucidation [98,99]. For instance, the *BnSHP1A09* gene has been confirmed to be associated with the regulation of lignin content; however, the synergistic mechanisms with other homologous genes remain unclear [98]. Moreover, the regulatory role of the miR319-TCP-FUL signaling pathway in silique development reveals a new molecular network, but the expression differences among various canola varieties and their interactions with environmental factors still need to be investigated in greater depth [100]. Future research can further explore the key genes and regulatory networks associated with silique dehiscence through multi-omics integrative analysis (such as transcriptomics, proteomics, and metabolomics), providing more targets for breeding. In terms of genetic improvement, the use of genome editing technologies such as CRISPR-Cas9 for precise editing of target genes has shown significant effects. For instance, the successful knockout of the homologous gene *BnJAG* has notably enhanced the crack resistance of pods [99]. However, optimizing gene editing strategies without compromising other agronomic traits (such as seed yield and quality) remains a focal point for future research. Additionally, utilizing QTL mapping and genome-wide association studies (GWAS) to identify new loci for crack resistance, in conjunction with molecular marker-assisted selection (MAS) techniques, is expected to accelerate the breeding process for crack-resistant varieties [101]. In biomechanical research, the crack resistance of pods is closely related to their morphological structure (such as pod length, width, and thickness) and mechanical properties (such as bending strength and crack resistance index) [34,]. Future studies may integrate high-precision imaging technologies and computer simulations to provide a more accurate analysis of the stress distribution and cracking mechanisms of pods. For example, finite element analysis (FEA) can simulate the stress distribution of pods under various mechanical forces, providing a theoretical basis for optimizing the design of harvesting machinery []. Furthermore, investigating the changes in the mechanical properties of pods under different environmental conditions (such as humidity and temperature) will aid in formulating more precise harvesting strategies [34]. In terms of cultivation management, optimizing agronomic measures such as nitrogen fertilizer application, planting density, and irrigation strategies can effectively enhance the crack resistance of pods. For instance, research indicates that a reasonable increase in nitrogen fertilizer application and the optimization of fertilization ratios (such as the ratio of base fertilizer to top dressing) can significantly improve both the yield and crack resistance of pods [102,103]. Additionally, optimizing planting density not only increases yield but also enhances the plant’s resistance to lodging, thereby indirectly improving the crack resistance of pods [104]. Future studies could further explore the interactive effects of different cultivation practices and environmental factors, providing a scientific basis for the development of region-specific cultivation management plans.

**Figure 3:**
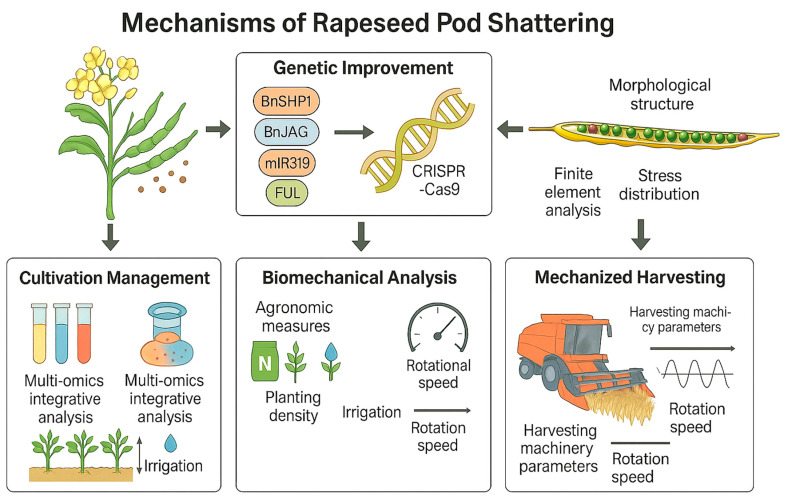
Future research directions of rapeseed pod cracking.

Finally, how to reduce the mechanical damage to siliques during the harvesting process is a key focus of future research in mechanized harvesting technology. For instance, by optimizing parameters such as the rotational speed of harvesting machinery, cutting height, and vibration frequency, the cracking rate of siliques can be effectively reduced [105]. Additionally, the development of novel crack-resistant materials and the design of more efficient harvesting devices are also important directions for future technological innovation (Figure 3).

In summary, future research on the pod shattering of rapeseed should integrate multidisciplinary approaches, including molecular biology, genetics, biomechanics, and agronomy. It should involve gene mining, genetic improvement, biomechanical analysis, cultivation management, and mechanized harvesting techniques, to comprehensively elucidate the mechanisms of pod shattering and develop efficient and precise anti-shattering strategies, thereby providing technical support for the sustainable development of rapeseed production.

## Data Availability

No new data were created or analyzed in this study.

## References

[1] Aquilia S., Bello C., Pinna M., Bianchi S., Giurlani W., Ciardelli F., Rosi L., Papini A.M. (2025). Incorporation of Protein Hydrolysate into Rapeseed Meal-Based Materials to Improve Flexibility. Polymers.

[2] Quan X., Wang X., Chen C., Ma T., Wang D., Zhang Y., Liu J. (2025). A comparative evaluation of using bio- and petroleum-based rejuvenators in aged asphalt rejuvenation: Rheological performance, microscopic properties, and life cycle assessments. Constr. Build. Mater..

[3] Witaszek K., Kupryaniuk K., Kupryaniuk J., Panasiewicz J., Czekala W. (2025). Optimization of Straw Particle Size for Enhanced Biogas Production: A Comparative Study of Wheat and Rapeseed Straw. Energies.

[4] Decsi K., Hegedus G., Kutasy B., Virag E. (2022). RNA-seq datasets of field rapeseed (*Brassica napus*) cultures conditioned by Elice16Indures biostimulator. Data Brief.

[5] MacIntosh S.C., Shaw M., Connelly M., Yao Z.J. (2021). Food and Feed Safety of NS-B5oo27-4 Omega-3 Canola (*Brassica napus*): A New Source of Long-Chain Omega-3 Fatty Acids. Front. Nutr..

[6] Tan Z.D., Han X., Dai C., Lu S.P., He H.Z., Yao X., Chen P., Yang C., Zhao L., Yang Q.Y. (2024). Functional genomics of *Brassica napus*: Progresses, challenges, and perspectives. J. Integr. Plant Biol..

[7] Yun J., Wang C., Zhang F., Chen L., Sun Z., Cai Y., Luo Y., Liao J., Wang Y., Cha Y. (2023). A nitrogen fixing symbiosis-specific pathway required for legume flowering. Sci. Adv..

[8] Watson C.A., Reckling M., Preissel S., Bachinger J., Bergkvist G., Kuhlman T., Lindstrom K., Nemecek T., Topp C.F.E., Vanhatalo A., Sparks D.L. (2017). Grain Legume Production and Use in European Agricultural Systems. Advances in Agronomy.

[9] Gao Z., Tu Y., Liao C., Guo P., Tian Y., Zhou Y., Xie Q., Chen G., Hu Z. (2024). Overexpression of *SlALC* Increases Drought and Salt Tolerance and Affects Fruit Dehiscence in Tomatoes. Int. J. Mol. Sci..

[10] Wang J., Wu X.F., Tang Y., Li J.G., Zhao M.L. (2021). RNA-Seq Provides New Insights into the Molecular Events Involved in “Ball-Skin versus Bladder Effect” on Fruit Cracking in Litchi. Int. J. Mol. Sci..

[11] Shirokova A.V., Volovik V.T., Zagoskina N.V., Zaitsev G.P., Khudyakova H.K., Korovina L.M., Krutius O.N., Nikolaeva T.N., Simonova O.B., Alekseev A.A. (2020). From Dimness to Glossiness-Characteristics of the Spring Rapeseed Mutant Form without Glaucous Bloom (*Brassica napus* L.). Agronomy.

[12] Karlsson I., Friberg H., Kolseth A.-K., Steinberg C., Persson P. (2017). Agricultural factors affecting *Fusarium* communities in wheat kernels. Int. J. Food Microbiol..

[13] Zumajo-Cardona C., Ambrose B.A., Madrigal Y., Pabon-Mora N. (2025). Dehiscent fruits in Brassicaceae and Papaveraceae: Convergent morpho-anatomical features with divergent underlying genetic mechanisms. Ann. Bot..

[14] Kanwal M., Rabbani A.M., Iqbal S., Fayyaz L., Afzal M. (2014). Genetic diversity in *Brassica* species AND *Eruca sativa* for yield associated parameters. Genet.

[15] Li Y.-L., Yu Y.-K., Zhu K.-M., Ding L.-N., Wang Z., Yang Y.-H., Cao J., Xu L.-Z., Li Y.-M., Tan X.-L. (2021). Down-regulation of *MANNANASE7 gene* in *Brassica napus* L. enhances silique dehiscence-resistance. Plant Cell Rep..

[16] Marsh J.I., Nestor B.J., Petereit J., Tay Fernandez C.G., Bayer P.E., Batley J., Edwards D. (2023). Legume-wide comparative analysis of pod shatter locus *PDH1* reveals phaseoloid specificity, high cowpea expression, and stress responsive genomic context. Plant J..

[17] Sun W., Chen Y., Zeng J., Li C., Yao M., Liu M., Ma Z., Huang L., Yan J., Zhan J. (2023). The Tartary buckwheat bHLH gene ALCATRAZ contributes to silique dehiscence in Arabidopsis thaliana. Plant Sci..

[18] Zhang T., Hong Y., Zhang X., Yuan X., Chen S. (2022). Relationship between Key Environmental Factors and the Architecture of Fruit Shape and Size in Near-Isogenic Lines of Cucumber (*Cucumis sativus* L.). Int. J. Mol. Sci..

[19] Pan Y., Luo Y., Bao J., Wu C., Wang J., Liu M., Yan F. (2024). Screening candidate genes for fruit size based on QTL-seq in Chinese jujube. Front. Plant Sci..

[20] Yu J., Zhu M., Bai M., Xu Y., Fan S., Yang G. (2020). Effect of calcium on relieving berry cracking in grape (*Vitis vinifera* L.) *‘Xiangfei’*. PeerJ.

[21] Ren H., Zhao Q., Feng Y., Tang P., Wang Y., Jiang J., Hu C., Wang Y., Cui B., Xie X. (2023). Gene-specific silencing of SlPL16, a pectate lyase coding gene, extends the shelf life of tomato fruit. Postharvest Biol. Technol..

[22] D’Aquino S., Strano M.C., Gentile A., Palma A. (2022). Decay Incidence and Quality Changes of Film Packaged ‘Simeto’ Mandarins Treated with Sodium Bicarbonate. Horticulturae.

[23] Yudina L., Sukhova E., Mudrilov M., Nerush V., Pecherina A., Smirnov A.A., Dorokhov A.S., Chilingaryan N.O., Vodeneev V., Sukhov V. (2022). Ratio of Intensities of Blue and Red Light at Cultivation Influences Photosynthetic Light Reactions, Respiration, Growth, and Reflectance Indices in Lettuce. Biology.

[24] Litt P.K., Kelly A., Omar A., Johnson G., Vinyard B.T., Kniel K.E., Sharma M. (2021). Temporal and Agricultural Factors Influence *Escherichia coli* Survival in Soil and Transfer to Cucumbers. Appl. Environ. Microbiol..

[25] Liu L., Javed H.H., Hu Y., Luo Y.-Q., Peng X., Wu Y.-C. (2024). Research progress and mitigation strategies for pod shattering resistance in rapeseed. PeerJ.

[26] Qing Y., Li Y., Xu L., Ma Z. (2021). Screen Oilseed Rape (*Brassica napus*) Suitable for Low-Loss Mechanized Harvesting. Agric.

[27] Chu W., Liu J., Cheng H., Li C., Fu L., Wang W., Wang H., Hao M., Mei D., Liu K. (2022). A lignified-layer bridge controlled by a single recessive gene is associated with high pod-shatter resistance in *Brassica napus* L.. Crop J..

[28] Hussain Q., Zhan J., Liang H., Wang X., Liu G., Shi J., Wang H. (2022). Key genes and mechanisms underlying natural variation of silique length in oilseed rape (*Brassica napus* L.) *germplasm*. Crop J..

[29] Liu J., Zhou R., Wang W., Wang H., Qiu Y., Raman R., Mei D., Raman H., Hu Q. (2020). A copia-like retrotransposon insertion in the upstream region of the *SHATTERPROOF1* gene, *BnSHP1*. A9, is associated with quantitative variation in pod shattering resistance in oilseed rape. J. Exp. Bot..

[30] Li H., Li F., Wang M., Hou C., Jia F., Wang X., Li M. (2025). Growth and selenium bioaccumulation in rape seedlings promoted by strain *Limosilactobacillus* sp. LF-17. BMC Plant Biol..

[31] Weymann W., Boettcher U., Sieling K., Kage H. (2015). Effects of weather conditions during different growth phases on yield formation of winter oilseed rape. Field Crops Res..

[32] Zheng M., Terzaghi W., Wang H., Hua W. (2022). Integrated strategies for increasing rapeseed yield. Trends Plant Sci..

[33] de Oliveira A.M.R.C.B., Yu P. (2023). Research progress and future study on physicochemical, nutritional, and structural characteristics of canola and rapeseed feedstocks and co-products from bio-oil processing and nutrient modeling evaluation methods. Crit. Rev. Food Sci. Nutr..

[34] Zhang M., Li G., Yang Y., Jin M., Wang G. (2023). Test Trials and Analysis of Pod-Shattering Characteristics of Harvested Rapeseed Silique. Appl. Sci..

[35] Li L., Li J., Wei C., Yang C., Zhong S. (2022). Effect of Mechanized Ridge Tillage with Rice-Rape Rotation on Paddy Soil Structure. Agric.

[36] Hu Q., Hua W., Yin Y., Zhang X., Liu L., Shi J., Zhao Y., Qin L., Chen C., Wang H. (2017). Rapeseed research and production in China. Crop J..

[37] Qian L., Lu H., Gao Q., Lu H.L. (2022). Household-owned farm machinery vs. outsourced machinery services: The impact of agricultural mechanization on the land leasing behavior of relatively large-scale farmers in China. Land Use Policy.

[38] Jiang T., Guan Z., Li H., Zhang M., Mu S., Wu C., Jin M. (2025). Collaborative optimization method of cleaning operational performance and multiparameter online control system for combine harvesters. Comput. Electron. Agric..

[39] Yang M., He J., Wan S., Li W., Chen W., Wang Y., Jiang X., Cheng P., Chu P., Shen W. (2021). Fine mapping of the *BnaC04*. BIL1 gene controlling plant height in Brassica napus L. BMC Plant Biol..

[40] Ping X., Ye Q., Yan M., Zeng J., Yan X., Li H., Li J., Liu L. (2022). Integrated genetic mapping and transcriptome analysis reveal the BnaA03. IAA7 protein regulates plant architecture and gibberellin signaling in Brassica napus L. Theor. Appl. Genet..

[41] Guo C.Y., Bai Z.H., Wang X.Z., Zhang W.S., Chen X.J., Lakshmanan P., Ma L., Lu J.W., Liu B., Shi X.J. (2022). Spatio-temporal assessment of greenhouse gas emission from rapeseed production in China by coupling nutrient flows model with LCA approach. Food Energy Secur..

[42] Kazemi H., Bourkheili S.H., Kamkar B., Soltani A., Gharanjic K., Nazari N.M. (2016). Estimation of greenhouse gas (GHG) emission and energy use efficiency (EUE) analysis in rainfed canola production (case study: Golestan province, Iran). Energy.

[43] Zhang Y.C., Mei H.C., Yan Z.H., Hu A.B., Wang S.M., Feng C.H., Chen K.H., Li W., Zhang X.H., Ji P.P. (2023). Year-Round Production of Cotton and Wheat or Rapeseed Regulated by Different Nitrogen Rates with Crop Straw Returning. Agronomy.

[44] Jung J., Maeda M., Chang A., Bhandari M., Ashapure A., Landivar-Bowles J. (2021). The potential of remote sensing and artificial intelligence as tools to improve the resilience of agriculture production systems. Curr. Opin. Biotechnol..

[45] Karunathilake E.M.B.M., Le A.T., Heo S., Chung Y.S., Mansoor S. (2023). The Path to Smart Farming: Innovations and Opportunities in Precision Agriculture. Agric.

[46] Zhan G.C., Zong W.Y., Ma L., Wei J.Y., Liu W. (2023). Biomechanical properties of ready-to-harvest rapeseed plants: Measurement and analysis. Inf. Process. Agric..

[47] Steponavicius D., Kemzuraite A., Bausa L., Zaleckas E. (2019). Evaluation of the Effectiveness of Pod Sealants in Increasing Pod Shattering Resistance in Oilseed Rape (Brassica napus L.). Energies.

[48] Li Q., Luo T., Cheng T., Yang S.T., She H.J., Li J., Wang B., Kuai J., Wang J., Xu Z.H. (2023). Evaluation and Screening of Rapeseed Varieties (*Brassica napus* L.) Suitable for Mechanized Harvesting with High Yield and Quality. Agronomy.

[49] Kowsalya K., Halka J., Anand M., Sahayarayan J.J., Rajkumar R., Arun M. (2025). Unraveling the multifaceted role of ethephon in plant physiology: From seed germination to crop maturation and harvesting. J. Plant Biochem. Biotechnol..

[50] Wang C.M., Xu M.C., Wang Y.C., Batchelor W.D., Zhang J., Kuai J., Ling L. (2022). Long-Term Optimal Management of Rapeseed Cultivation Simulated with the CROPGRO-Canola Model. Agronomy.

[51] Zhang Z., Cong R.H., Ren T., Li H., Zhu Y., Lu J.W. (2020). Optimizing agronomic practices for closing rapeseed yield gaps under intensive cropping systems in China. J. Integr. Agric..

[52] Lou H.X., Zhao B.W., Peng Y., El-Badri A.M., Batool M., Wang C.Y., Wang Z.K., Huang W., Wang T.Y., Li Z. (2023). Auxin plays a key role in nitrogen and plant density-modulated root growth and yield in different plant types of rapeseed. Field Crops Res..

[53] Raman H., Raman R., Sharma N., Cui X.B., McVittie B., Qiu Y., Zhang Y.Y., Hu Q., Liu S.Y., Gororo N. (2023). Novel quantitative trait loci from an interspecific *Brassica rapa* derivative improve pod shatter resistance in *Brassica napus*. Front. Plant Sci..

[54] Feng T., Qiu Z.P., Wang H. (2024). Tipping points in seed dispersal mutualism driven by environmental stochasticity. Siam J. Appl. Math..

[55] Zhang Y., Shen Y.Y., Wu X.M., Wang J.B. (2016). The basis of pod dehiscence: Anatomical traits of the dehiscence zone and expression of eight pod shatter-related genes in four species of *Brassicaceae*. Biol. Plant..

[56] Zhang W., Cao H., Zhang W., Liu Y., Ge D., Feng C., Chen W., Song C., Sijun G., Zhang Q. (2018). Biomass-Based Rapeseed (*Brassica napus*) Pod Morphological Model. Int. J. Agric. Biol..

[57] Tang M., Tong C., Liang L., Du C., Zhao J., Xiao L., Tong J., Dai X., Helal M.M.U., Dai W. (2020). A recessive high-density pod mutant resource of *Brassica napus*. Plant Sci..

[58] Mahmood U., Li X., Qian M., Fan Y., Yu M., Li S., Shahzad A., Qu C., Li J., Liu L. (2023). Comparative transcriptome and co-expression network analysis revealed the genes associated with senescence and polygalacturonase activity involved in pod shattering of rapeseed. Biotechnol. Biofuels Bioprod..

[59] Zhang F.G., Liu N., Chen T.H., Xu H., Li R., Wang L.Y., Zhou S., Cai Q.A., Hou X.Z., Wang L. (2024). Genome-wide identification of *GH28* family and insight into its contributions to pod shattering resistance in *Brassica napus* L. *BMC Genom*. BMC Genom..

[60] Wang D., Lu Q., Jin S., Fan X., Ling H. (2023). Pectin, Lignin and Disease Resistance in *Brassica napus* L.: An Update. Horticulturae.

[61] Liljegren S.J., Ditta G.S., Eshed Y., Savidge B., Bowman J.L., Yanofsky M.F. (2000). SHATTERPROOF MADS-box genes control seed dispersal in Arabidopsis. Nature.

[62] Zhai Y., Cai S., Hu L., Yang Y., Amoo O., Fan C., Zhou Y. (2019). CRISPR/Cas9-mediated genome editing reveals differences in the contribution of INDEHISCENT homologues to pod shatter resistance in Brassica napus L.. Theor. Appl. Genet..

[63] Ohama N., Moo T.L., Chung K., Mitsuda N., Boonyaves K., Urano D., Chua N.-H. (2025). MEDIATOR15 destabilizes DELLA protein to promote gibberellin-mediated plant development. New Phytol..

[64] Lynch T.J., Erickson McNally B.J., Losic T., Lundquist J., Finkelstein R. (2024). ABI5 Binding Proteins are substrates of key components in the ABA core signaling pathway. bioRxiv.

[65] Roberts J.A., Elliott K.A., Gonzalez-Carranza Z.H. (2002). Abscission, dehiscence, and other cell separation processes. Annu. Rev. Plant Biol..

[66] Patharkar O.R., Walker J.C. (2019). Connections between abscission, dehiscence, pathogen defense, drought tolerance, and senescence. Plant Sci..

[67] Afridi M., Ahmad K., Malik S.S., Rehman N., Yasin M., Khan S.M., Hussain A., Khan M.R. (2022). Genome-wide identification, phylogeny, and expression profiling analysis of shattering genes in rapeseed and mustard plants. J. Genet. Eng. Biotechnol..

[68] Stephenson P., Stacey N., Bruser M., Pullen N., Ilyas M., O’Neill C., Wells R., Ostergaard L. (2019). The power of model-to-crop translation illustrated by reducing seed loss from pod shatter in oilseed rape. Plant Reprod..

[69] Braatz J., Harloff H.-J., Mascher M., Stein N., Himmelbach A., Jung C. (2017). CRISPR-Cas9 Targeted Mutagenesis Leads to Simultaneous Modification of Different Homoeologous Gene Copies in Polyploid Oilseed Rape (*Brassica napus*). Plant Physiol..

[70] Cui Y., Su Y., Bian J., Han X., Guo H., Yang Z., Chen Y., Li L., Li T., Deng X.W. (2024). Single-nucleus RNA and ATAC sequencing analyses provide molecular insights into early pod development of peanut fruit. Plant Commun..

[71] Guo M.W., Zhu L., Li H.Y., Liu W.P., Wu Z.N., Wang C.H., Liu L., Li Z.Y., Li J. (2022). Mechanism of pod shattering in the forage legume *Medicago ruthenica*. Plant Physiol. Biochem..

[72] Li S., Wang W., Sun L., Zhu H., Hou R., Zhang H., Tang X., Clark C.B., Swarm S.A., Nelson R.L. (2024). Artificial selection of mutations in two nearby genes gave rise to shattering resistance in soybean. Nat. Commun..

[73] Islam M.T., Liu Y., Hassan M.M., Abraham P.E., Merlet J., Townsend A., Jacobson D., Buell C.R., Tuskan G.A., Yang X. (2024). Advances in the Application of Single-Cell Transcriptomics in Plant Systems and Synthetic Biology. Biodesign Res..

[74] Mukhtiar A., Ullah S., Yang B., Jiang Y.-Q. (2025). Unlocking genetic potential: A review of the role of CRISPR/Cas technologies in rapeseed improvement. Stress Biol..

[75] Jedlickova V., Hejret V., Demko M., Jedlicka P., Stefkova M., Robert H.S. (2023). Transcriptome analysis of thermomorphogenesis in ovules and during early seed development in *Brassica napus*. BMC Genom..

[76] Khan D., Ziegler D.J., Kalichuk J.L., Hoi V., Huynh N., Hajihassani A., Parkin I.A.P., Robinson S.J., Belmonte M.F. (2022). Gene expression profiling reveals transcription factor networks and subgenome bias during *Brassica napus* seed development. Plant J..

[77] Han X., Peng Y., Yin S., Zhao H., Zong Z., Tan Z., Zhang Y., Ma W., Guo L. (2024). Transcriptional regulation of transcription factor genes WRI1 and LAFL during Brassica napus seed development. Plant Physiol..

[78] Ziegler D.J., Khan D., Pulgar-Vidal N., Parkin I.A.P., Robinson S.J., Belmonte M.F. (2023). Genomic asymmetry of the *Brassica napus* seed: Epigenetic contributions of DNA methylation and small RNAs to subgenome bias. Plant J..

[79] Khan M.H.U., Hu L., Zhu M., Zhai Y., Khan S.U., Ahmar S., Amoo O., Zhang K., Fan C., Zhou Y. (2021). Targeted mutagenesis of *EOD3* gene in *Brassica napus* L. regulates seed production. J. Cell. Physiol..

[80] Tan H., Zhang J., Qi X., Shi X., Zhou J., Wang X., Xiang X. (2019). Correlation analysis of the transcriptome and metabolome reveals the regulatory network for lipid synthesis in developing *Brassica napus* embryos. Plant Mol. Biol..

[81] Zhang C., Chang W., Li X., Yang B., Zhang L., Xiao Z., Li J., Lu K. (2022). Transcriptome and Small RNA Sequencing Reveal the Mechanisms Regulating Harvest Index in *Brassica napus*. Front. Plant Sci..

[82] Nichol J.B., Samuel M.A. (2024). Characterizing the role of endocarp *a* and *b* cells layers during pod (silique) development in Brassicaceae. Plant Signal. Behav..

[83] Liu D., Wu J., Lin L., Li P., Li S., Wang Y., Li J., Sun Q., Liang J., Wang Y. (2021). Overexpression of *Cinnamoyl-CoA Reductase 2* in *Brassica napus* Increases Resistance to *Sclerotinia sclerotiorum* by Affecting Lignin Biosynthesis. Front. Plant Sci..

[84] Jiang J., Liao X., Jin X., Tan L., Lu Q., Yuan C., Xue Y., Yin N., Lin N., Chai Y. (2020). MYB43 in Oilseed Rape (*Brassica napus*) Positively Regulates Vascular Lignification, Plant Morphology and Yield Potential but Negatively Affects Resistance to *Sclerotinia sclerotiorum*. Genes.

[85] Lin N., Wang M., Jiang J., Zhou Q., Yin J., Li J., Lian J., Xue Y., Chai Y. (2023). Downregulation of *Brassica napus MYB69 (BnMYB69*) increases biomass growth and disease susceptibility via remodeling phytohormone, chlorophyll, shikimate and lignin levels. Front. Plant Sci..

[86] Yin N., Li B., Liu X., Liang Y., Lian J., Xue Y., Qu C., Lu K., Wei L., Wang R. (2022). Two types of cinnamoyl-CoA reductase function divergently in accumulation of lignins, flavonoids and glucosinolates and enhance lodging resistance in *Brassica napus*. Crop J..

[87] Hou J., Riaz M., Yan L., Lu K., Jiang C. (2022). Effect of exogenous L-aspartate nano-calcium on root growth, calcium forms and cell wall metabolism of *Brassica napus* L. *Nanoimpact*
**2022**, *27*, 100415. Nanoimpact.

[88] Wang D., Jin S., Chen Z., Shan Y., Li L. (2022). Genome-wide identification of the pectin methylesterase inhibitor genes in *Brassica napus* and expression analysis of selected members. Front. Plant Sci..

[89] Dou Y., Yang Y., Mund N.K., Wei Y., Liu Y., Wei L., Wang Y., Du P., Zhou Y., Liesche J. (2021). Comparative Analysis of Herbaceous and Woody Cell Wall Digestibility by Pathogenic Fungi. Molecules.

[90] Xu B., Gong X., Chen S., Hu M., Zhang J., Peng Q. (2021). Transcriptome Analysis Reveals the Complex Molecular Mechanisms of *Brassica napus*-*Sclerotinia sclerotiorum* Interactions. Front. Plant Sci..

[91] Rashid M., Hampton J.G., Shaw M.L., Rolston M.P., Khan K.M., Saville D.J. (2020). Oxidative damage in forage rape (*Brassica napus* L.) seeds following heat stress during seed development. J. Agron. Crop Sci..

[92] Wang J., Fan Y., Mao L., Qu C., Lu K., Li J., Liu L. (2021). Genome-wide association study and transcriptome analysis dissect the genetic control of silique length in *Brassica napus* L. *Biotechnol*. Biofuels.

[93] Yan J., Liao X., He R., Zhong M., Feng P., Li X., Tang D., Liu X., Zhao X. (2017). Ectopic expression of GA 2-oxidase 6 from rapeseed (*Brassica napus* L.) causes dwarfism, late flowering and enhanced chlorophyll accumulation in *Arabidopsis thaliana*. Plant Physiol. Biochem..

[94] Zhao M., Li J., Zhou S., Li K., Niu L., Zhao L., Xu D. (2023). Analysis of the effects of sulfamethoxazole on the secondary metabolites and antioxidants in oilseed rape (*Brassica napus* L.) *and the underlying mechanisms*. Sci. Total Environ..

[95] Wang L., Wang R., Lei W., Wu J., Li C., Shi H., Meng L., Yuan F., Zhou Q., Cui C. (2021). Transcriptome analysis reveals gene responses to herbicide, tribenuron methyl, in *Brassica napus* L. during seed germination. BMC Genom..

[96] Shahid M., Cai G., Zu F., Zhao Q., Qasim M.U., Hong Y., Fan C., Zhou Y. (2019). Comparative Transcriptome Analysis of Developing Seeds and Silique Wall Reveals Dynamic Transcription Networks for Effective Oil Production in *Brassica napus* L.. Int. J. Mol. Sci..

[97] Liu Y., Hua Y.-p., Chen H., Zhou T., Yue C.-p., Huang J.-y. (2021). Genome-scale identification of *plant defensin* (*PDF*) family genes and molecular characterization of their responses to diverse nutrient stresses in allotetraploid rapeseed. PeerJ.

[98] Zaman Q.U., Chu W., Shi Y., Hao M., Mei D., Jacqueline B., Zhang B., Li C., Hu Q. (2021). Characterization of *SHATTERPRROOF* Homoeologs and CRISPR-Cas9-Mediated Genome Editing Enhances Pod-Shattering Resistance in *Brassica napus* L.. Cris. J..

[99] Zaman Q.U., Chu W., Hao M., Shi Y., Sun M., Sang S.-F., Mei D., Cheng H., Liu J., Li C. (2019). CRISPR/Cas9-Mediated Multiplex Genome Editing of JAGGED Gene in *Brassica napus* L. *Biomolecules*
**2019**, *9*, 725. Biomolecules.

[100] Cao B., Wang H., Bai J., Wang X., Li X., Zhang Y., Yang S., He Y., Yu X. (2022). *miR*319-Regulated TCP3 Modulates Silique Development Associated with Seed Shattering in Brassicaceae. Cells.

[101] Raman R., Qiu Y., Coombes N., Raman H. (2025). Identification and validation of genomic regions for pod shatter resistance in *Brassica rapa* using QTL-seq and traditional QTL mapping. Bmc Plant Biol..

[102] Zuo Q., Liu J., Wang L., Yang G., Leng S. (2020). Yield, dry matter and N characteristics in canola as affected by fertilizer N rate and split-application ratio under high soil fertility condition. J. Plant Nutr..

[103] Dogra P., Thakur A., Kukreja S., Alfagham A.T., Gupta R.K., Ahmad M., Dahiya Y., Siddiqui M.H., Alamri S. (2025). Synergistic Effects of Salicylic Acid, Hydrogel, and Sulphur Sources for Boosting the Yield of Rapeseed under Limited Irrigation. Bioresources.

[104] Wang R., Wu W., Cheng X., Peng W. (2023). High plant density increases sunlight interception and yield of direct-seeded winter canola in China. Exp. Agric..

[105] Zhang M., Li G., Yang Y., Jin M., Jiang T. (2023). Design and Parameter Optimization of Variable Speed Reel for Oilseed Rape Combine Harvester. Agric.

